# Confirming Multiplex RT-qPCR Use in COVID-19 with Next-Generation Sequencing: Strategies for Epidemiological Advantage

**DOI:** 10.1155/2022/2270965

**Published:** 2022-07-30

**Authors:** Rob E. Carpenter, Vaibhav Tamrakar, Harendra Chahar, Tyler Vine, Rahul Sharma

**Affiliations:** ^1^Advanta Genetics, 10935 CR 159, Tyler, TX 75703, USA; ^2^University of Texas at Tyler, 3900 University Boulevard, Tyler, TX 75799, USA; ^3^ICMR-National Institute of Research in Tribal Health, Jabalpur, MP 482003, India; ^4^RetroBioTech LLC, Coppell, TX 75019, USA; ^5^University of Miami Miller School of Medicine, 1600 NW 10th Ave, Miami, FL 33136, USA

## Abstract

Rapid identification and tracking of emerging SARS-CoV-2 variants are critical for understanding the transmission dynamics and developing strategies for interrupting the transmission chain. Next-Generation Sequencing (NGS) is an exceptional tool for whole-genome analysis and deciphering new mutations. The technique has been instrumental in identifying the variants of concern (VOC) and tracking this pandemic. However, NGS is complex and expensive for large-scale adoption, and epidemiological monitoring with NGS alone could be unattainable in limited-resource settings. In this study, we explored the application of RT-qPCR-based detection of the variant identified by NGS. We analyzed a total of 78 deidentified samples that screened positive for SARS-CoV-2 from two timeframes, August 2020 and July 2021. All 78 samples were classified into WHO lineages by whole-genome sequencing and then compared with two commercially available RT-qPCR assays for spike protein mutation(s). The data showed good concordance between RT-qPCR and NGS analysis for specific SARS-CoV-2 lineages and characteristic mutations. RT-qPCR assays are quick and cost-effective and thus can be implemented in synergy with NGS for screening NGS-identified mutations of SARS-CoV-2 for clinical and epidemiological interest. Strategic use of NGS and RT-qPCR can offer several COVID-19 epidemiological advantages.

## 1. Introduction

The coronavirus (COVID-19) pandemic started in December 2019 in Wuhan, China. It has been considered one of the deadliest infectious disease outbreaks in recent world history. The causative agent of COVID-19 is the Severe Acute Respiratory Syndrome Coronavirus 2 (SARS-CoV-2). SARS-CoV-2 is a positive-sense RNA virus belonging to the Coronaviridae family, genus *Betacoronavirus*, and subgenus *sarbecovirus* [1, 2]. Coronavirus has had devastating effects on the human population and to date is estimated to have caused over 5 million deaths worldwide [3]. Rapid accurate diagnosis of SARS-CoV-2 is the most crucial step in the management of COVID-19—mostly achieved with reverse transcription-quantitative polymerase chain reaction (RT-qPCR). The assays detect highly conserved regions in the open reading frame (ORF) 1a or 1b and the nucleocapsid (*N*) gene of SARS-CoV-2 [4–6].

Currently, the virus continues to be a global agent of infection. The highly mutagenic nature of SARS-CoV-2 has assaulted many countries with second or third waves of the outbreak [7, 8]. Mutations with higher transmissibility, a more intense disease state, and that are less likely to respond to vaccines or treatments, have been classified by the World Health Organization (WHO) as variants of concern (VOC; Table 1). Recent epidemiological reports released by WHO indicated five VOCs: (1) Alpha (B.1.1.7), first reported in the United Kingdom (UK) in December 2020, (2) Beta (B.1.351), first reported in South Africa in December 2020, (3) Gamma (P.1), first reported in Brazil January 2021, (4) Delta (B.1.617.2), first reported in India December 2020, and (5) Omicron (B.1.1.529) reported from multiple countries (Cascella et al.) [7] Genomic changes in the receptor-binding domain (RBD), a region of the spike protein that studs SARS-CoV-2 to the outer cell surface, are linked to increased capacity to strike in several outbreak phases in different parts of the world [9]. More recently, South Africa reported a new SARS-CoV-2 variant to the WHO. Omicron (B.1.1.529) was first detected in specimens collected in Botswana. On November 26, 2021, the Technical Advisory Group on SARS-CoV-2 Virus Evolution (TAG-VE) advised WHO to designate B.1.1.529 as the fifth VOC [10].

There continues to be a need for swift and cost-effective SARS-CoV-2 variant detection and monitoring. Genomic sequencing is the gold standard and most reliable method for the detection of such changes in the viral genome. The standard Sanger sequencing method is highly accurate but it can only sequence a small fraction of the genome [12]. Sanger sequencing is also laborious, time-consuming, and expensive for large-scale sequencing projects that require rapid turnaround times. These attributes make Sanger sequencing less attractive for SARS-CoV-2 sequencing for variant identification and monitoring.

Targeted Next-Generation Sequencing (NGS) is also a reliable method to identify variant strains of pathogens, including viruses [13]. The principal advantage of NGS over other techniques like Sanger sequencing or RT-qPCR is that scientists and laboratorians do not require prior knowledge of existing nucleotide sequences. Moreover, NGS has higher discovery power and higher throughput [13]. In the current pandemic, NGS has widely been employed to detect and identify novel mutated viral variants of SARS-CoV-2 [14]. Although widespread adoption of NGS in clinical laboratories offers effective variant discovery, several challenges impede the routine use of NGS in these settings. Besides the need for multifaceted NGS validation studies [15], NGS testing is complicated by the high level of necessary human expertise and the higher cost of scalability for routine pathogen/variant detection. Moreover, the interpretation of results generated by NGS can be intricately complex and their applicability to clinical decision-making is another issue altogether. These complexities pose the need to progress practical methodologies to identify SARS-CoV-2 mutagenic variants quickly and cost-effectively.

PCR is the gold standard for the detection of preidentified genomic sequences and variations. Several RT-qPCR tests were developed and commercialized very quickly after the first SARS-CoV-2 genome was sequenced. Technology has proven to be the most reliable tool for the diagnosis of COVID-19 and was adopted globally because of its lower cost and complexity. Likewise, RT-qPCR can be deployed for mass-scale detection of a new mutation after it is discovered by NGS. This approach has been widely deployed in environmental surveillance of known COVID-19 variants, and RT-qPCR is reported as a gold standard test for COVID-19 surveillance in wastewater. Droplet digital PCR (ddPCR) is a modified PCR method where amplification is performed in submicroliter droplets, and the number of positive droplets is enumerated for absolute quantification of the targets or the genomic variations. ddPCR provides the absolute quantification of targets and is also reported to be more sensitive for the detection of known SARS-CoV-2 mutations [16].

Various variant-specific RT-qPCR assays have been developed. For example, spike protein mutation (L452R) is a characteristic mutation of the Delta variant, and several multiplex mutation-specific RT-qPCR assays have been developed to detect VOCs via NGS-identified mutations. RT-qPCR assays are widely adopted because of their lower turnaround time (<24 hrs). Capital investment and operational cost of RT-qPCR are also significantly cheaper than NGS [17]. In the United States, RT-qPCR-based COVID-19 testing is reimbursed at ∼$100/sample, compared to $300–$1000/sample for COVID-19 sequencing. NGS cost is highly dependent on the sample volume and instrument throughput. To achieve the lowest published cost, the laboratory has to sequence ∼30,000 samples in a single batch on a Million Dollar instruments.

Recent advances in RT-qPCR instruments have led to the development of smaller, cheaper, and portable instruments, and the COVID-19 pandemic has boosted the adoption of such instrumentation. Bechtold et al. have reported the development of RT-qPCR assays for the detection of VOCs on the basis of single nucleotide polymorphisms (N501Y, E484K, and deletion HV69/70) in spike protein. This assay is also validated for the field application using a portable peakPCR [18].

According to a technical report published by the European Centre for Disease Prevention and Control, the WHO Regional Office for Europe-related variant detection methods suggests NGS should perform for the confirmation of the newly emerged VOCs not for detection and prevalence calculating variants [19].

As soon as new mutations are discovered by NGS, academic and commercial researchers have rushed to design qPCR assays for the detection of the same mutation [20]. Thermo Fisher Scientific has developed mutation-specific assays as mutations are discovered. Several other companies (GT Molecular, PerkinElmer, Promega, and Twist Biosciences) developed multiplex RT-qPCR panels targeting mutations characteristic of the variant. Combinations of such reactions are available in kit format for the detection of known mutations defining the variants. These kits are widely used for SARS-CoV-2-variants surveillance in the environmental samples, such as wastewater [21].

We have compared the variant detection by two commercially available RT-qPCR-based solutions to whole-genome sequencing. The adoption of these kits in clinical surveillance has been restricted because of the limited clinical utility for individual patient variant identification. RT-qPCR-based variant detection is based on limited known mutations, compared to a whole-genome analysis by NGS which can identify known mutations as well as discover new mutations.

With the acknowledgment of these limitations, the current study proposes that RT-qPCR could be utilized to extend the mass scale detection of the mutation(s) discovered by NGS. Strategic deployment of NGS for discovering new mutations followed by mass surveillance by RT-qPCR could improve the epidemiological surveillance of this pandemic. Rapid detection of known variants could also potentially have a clinical application if future variants with different clinical manifestations and treatment needs are discovered.

## 2. Methodology

### 2.1. Sample Collection

This study used 78 deidentified sample remnants from nasopharyngeal or oropharyngeal swabs (catalog# 202003, Nest Biotechnology Jiangsu, China) collected from patients that screened positive for the presence of SARS-CoV-2 following RNA extraction and RT-qPCR at Advanta Genetics (https://aalabs.com/) in Tyler, Texas. All the clinical samples were collected from Texas residents who tested positive for SARS-CoV-2 with established protocols targeting N1 and N2 genes with established primer and probe design. Eleven samples were collected and archived during the early (August 2020) pandemic. The remaining 67 samples were collected in July 2021 following the global outbreak of the Delta (B.1.617.2) variant. We qualified the samples with moderate to high viral load by cycle threshold (Ct) values ≤30 for N1 and N2 genes by RT-qPCR testing on the LightCycler® 480 System (Roche). We also included 4 samples with low viral amplification (Ct = 30–35; sample 210–213) in the study to evaluate the applicability of RT-qPCR and NGS for reduced Ct values (Ct values are inversely proportional to amplification thresholds; Table 2). Written consent was obtained from the patients for participating in the study, and only residual diagnostic samples were used.

### 2.2. RNA Extraction

Total RNA was extracted from nasopharyngeal or oropharyngeal swabs collected and transported to the lab in MANTACC Transport Medium or Viral Transport Medium (VTM) purchased from Criterion Clinical (https://criterionclinical.com/). RNA extraction was carried out in a preamplification environment within a Biosafety level 2 (BSL-2) facility using the Roche MagNA Pure 96 System and Viral NA Small Volume Kits. Briefly, samples were lysed with 340 uL of lysis buffer and 10 uL of proteinase *K* at 55°C for 10 minutes, followed by extraction via the Roche MagNA Pure 96 instrument. Extracted nucleic acids were immediately sealed with a PCR clean sealing film (Cat #T329-1 Simport Scientific Inc. QC J3G 4S5 Canada) and frozen at −80°C until sequencing was imminent.

### 2.3. Library Preparation and Sequencing

Samples were sequenced in two laboratories using the Illumina Sequencing platform, 67 samples were sequenced at Fulgent Genetic (https://www.fulgentgenetics.com), and the remaining 21 samples were sequenced at Advanta Genetics. Sequencing libraries were prepared using the Illumina COVIDSeq protocol (Illumina Inc, USA). Total RNA was primed with random hexamers, and first-strand cDNA was synthesized using reverse transcriptase. The SARS-CoV-2 genome was amplified using the two sets of primers to produce amplicons spanning the entire genome of SARS-CoV-2. The amplified product was then processed for tagmentation and adapter ligation using 24 IDT for Illumina Nextera UD Indexes Set *A*. Further cleanup and pooling were performed as per protocols provided by the manufacturer (Illumina Inc, USA). A COVIDSeq positive control (Wuhan-Hu-1) and one no template control (NTC) were processed with each library batch. Representative libraries were quantified using a Qubit 2.0 fluorometer (Invitrogen, Inc.), and fragment sizes were analyzed in Agilent 5200 Fragment Analyzer. Libraries were pooled into an equimolar concentration, and the pool was further normalized to 1nM concentration. The final library pool was denatured and neutralized with 0.2N NaOH and 400 mM Tris-HCL (pH-8), respectively. Denatured libraries were further diluted to a 2 pm loading concentration. Dual indexed paired-end sequencing with 75 bp read length was carried out using the HO flow cell (150 cycles) on the Illumina MiniSeq® instrument.

### 2.4. NGS Data Analysis

Illumina BaseSpace (https://basespace.illumina.com) bioinformatics pipeline was used for sequencing QC, FASTQ Generation, genome assembly, and identification of SARS-CoV-2 variants. Briefly, the Binary Base Call (BCL) raw sequencing files generated by Illumina MiniSeq® sequencing platforms were uploaded to the Illumina BaseSpace online portal and demultiplexed to FASTQ format using the FASTQ Generation (Version: 1.0.0.) application. The raw FASTQ files were trimmed, sorted, and checked for quality (*Q* > 30) using the FASTQ-QC application within the BaseSpace. QC passed FASTQ files were aligned against the SARS-CoV-2 reference genome (NCBI RefSeq NC_045512.2) using Bio-IT Processor (Version: 0x04261818). Then, DRAGEN COVID Lineage (Version: 3.5.4) application in BaseSpace was used to generate a single consensus FASTA file for all the samples sequenced on a single flow cell. Finally, single consensus FASTQ was also analyzed for lineage assignment using the web version of Phylogenetic Assignment of Named Global Outbreak Lineages (PANGOLIN) software (https://pangolin.cog-uk.io). Only the consensus variants identified by both applications were used for further analysis.

### 2.5. Phylogenic Analysis

The FASTQ sequence file was analyzed and visualized for evolutionary relationships through the open-source toolkit Nextstrain (https://clades.nextstrain.org/). GSAID database for global SARS-CoV-2 sequence analysis, available from the Nexstrain server, was used to retrieve representative variant sequences [22]. The NCBI databank was used to retrieve the original Wuhan strain SARS-CoV-2 sequence. All the individual consensus genome sequence files were aligned by using the Clustal-W multiple sequence alignment tool [23]. The phylogenetic analysis was carried out utilizing the Clustal omega server and the phylogenetic tree was constructed using the Mega *X* tool [24] with default parameters of the maximum likelihood method.

**Figure 1:**
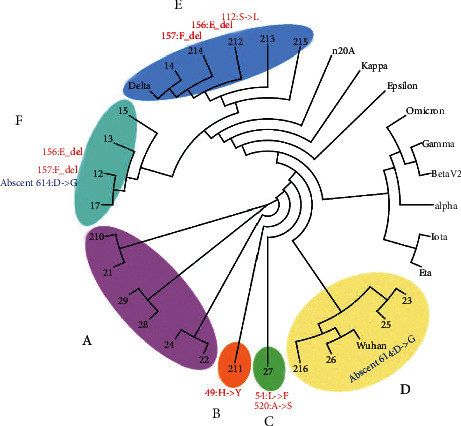
Minimum spanning tree of the SARS-CoV-2 variants identified in samples (*n* = 21) collected in August 2020 and July 2021.

The further analysis aimed at investigating the conservation of spike protein in reference sequences versus clinical strains of SARS-CoV-2 from our study using bioinformatics tools. The protein sequences for different ORFs were determined by either annotation by IBM Functional Genomics Platform. [25] T-COFFEE and PRALINE software [26, 27] were used for the alignment of spike proteins from different isolates and mutation position analysis (Figure 1).

### 2.6. COVID-19 Lineage Assignment Using RT-qPCR

Commercially available assays from two vendors (GT Molecular [Colorado USA], and Thermo Fisher Scientific [Massachusetts, USA]) were evaluated for detection of known variants, and results were compared to the NGS-based variant detection of the same samples (Table 3). Assays from GT Molecular detected 7 spike protein mutations (N501Y, Del69-70, E484K, K417N, K417T, L452R, and T478K).

GT Molecular assays were provided in two different kits containing the variant-specific reference standard and mutation-specific primer-probe. Amplifications are performed according to the manufacturer's instructions in separate master mix preparations as described in Table 3. Briefly, RNA was reverse transcribed for 10 minutes at 53°C followed by enzyme activation for 2 minutes at 95°C, and 40 40 cycles of 15 seconds at 95°C for Denaturation and 60 seconds at 52°C for Annealing/Extension. Reactions were performed by using qScript 1-Step Virus ToughMix (Quantabio Inc, Beverly, MA USA) on LightCycler® 480 System (Roche).

Two TaqMan assays from Thermo Fisher Scientific targeting two spike protein mutations (L452R and P681R) were performed according to the manufacturer's instructions on LightCycler® 480 System (Roche). The Delta variant classifying mutation (L452R) was used for the final classification of the Delta variant across all the RT-qPCR-based methods evaluated in this study. Whole-genome sequencing followed PANGOLIN classification and was used as the gold standard for the final variant classification and validation of the RT-qPCR-based variant detection methods. Unfortunately, we could not obtain the recent Omicron variant samples to extend our investigations to this recent variant.

## 3. Results

The 78 randomly selected positive SARS-CoV-2 samples were from two separate periods in the pandemic. NGS of the 11 samples from August 2020 revealed eight different lineages, but none of the lineages were VOC according to the WHO classification (Figure 1). All samples (100% [67/67]) sequenced from July 2020 revealed the SARS-CoV-2 Delta (B.1.617.2, AY3, and AY 25) VOC with sublineages AY.1 to AY.3. Incidentally, the six samples concurrently sequenced at both laboratories were identified as Delta (B.1.617.2) VOC. Unfortunately, raw data (FASTQ files) were not available from the samples sequenced at Fulgent Genetics. However, the raw data from the 21 samples sequenced at Advanta Genetics was analyzed for phylogenetic relationship and mutation discovery (Table 2). This data revealed novel mutations belonging to existing prominent lineages along with convergent mutations of different lineages and one unique mutation Figure 1.

*Note*. No Brazilian or United Kingdom lineages were identified. Two groups of samples (*F* & *D*) lacked omnipresent mutation (614: *D*->*G*) which is present in most variants of concern. Group *F* is of particular interest as it had most of the Delta variant mutations except 614: *D*->*G* which is present in all the samples unless otherwise marked. Group *E*, on the other hand, is Delta lineage with a novel mutation among them (i.e., 112: *S*->*L*). Group *A* was identical to the Wuhan strain except for the 614: *D*->*G* mutation. Two samples are carrying unique mutation sets: Set *B* had a single sample with mutation 49: *H*->*Y* which is novel and not found in any other lineage and Set *C* also had a single sample with unreported mutation pair 54: *L*->*F* and 520: *A*->*S*.

We then turned our focus to testing the 67 Delta (B.1.617.2) samples by using RT-qPCR methodology targeting three (L452R, T478K, and P681R) characteristic mutations identified through sequencing. We tested each sample using two different commercially available (Thermo Fisher Scientific and GT Molecular) assays and compared the results. The Delta (B.1.617.2) classifying mutation (L452R) was correctly identified by GT Molecular RT-qPCR-based assay, and the test showed 100% concordance for all 67 samples that were sequenced as Delta (B.1.617.2). However, the Thermo Fisher Scientific assay for the same target (L452R) did not amplify in 4 out of 67 samples otherwise identified as Delta variant by NGS (Table 3; Supplementary Table S1). All four samples had a relatively low viral load (Ct > 25), with overall higher Ct values for all the RT-qPCR assays. Considering the relatively low sensitivity of mutation-specific RT-qPCR compared to the target detection RT-qPCR in general and the lower sensitivity of Thermo Fisher Scientific assays, this slight discrepancy is not alarming. Overall, the GT Molecular assay targeting the L452R mutations had a 4.21 ± 2.3 lower Ct value when the same RNA template was tested with both assays suggesting higher sensitivity of the GT Molecular assay. Moreover, 5 of 67 samples were negative for T478K (GT Molecular), and 12/67 were negative for P681R specific PCR (Thermo Fisher Scientific) using RT-qPCR (Table 3). Unfortunately, we could not verify the absence of these mutations because NGS data was not available for the 67 samples sequenced at Fulgent Genetics. Thus, the L452R mutation remained the most informative marker for RT-qPCR-based detection of the Delta variant. All 11 samples sequenced as nonvariants of concern were negative for all three Delta variant-specific mutations (Table 3). Interestingly, a Beta and Gamma variant classifying mutation (E484K) was identified (both by RT-qPCR assays and NGS) in one sample, which is otherwise classified as a Delta variant by NGS and carries an L452R mutation. This mutation combination should be monitored and further investigated for its clinical significance.

Of note, the 4 samples with lower viral amplification (30 Ct) that were included in this study were able to be characterized by NGS and both RT-qPCR assays. Two out of the four samples were identified as Delta (B.1.617.2) variants with the remaining two identified as non-VOC. Therefore, NGS and RT-qPCR methodologies can potentially be used for SARS-CoV-2 variant detection from the samples with lower viral amplification (1000–10 copies).

**Figure 2:**
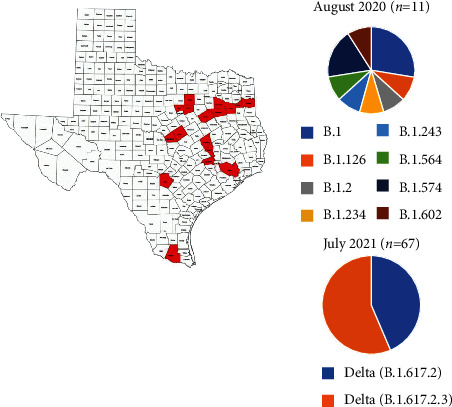
Evolution of the SARS-CoV-2 variant in Texas over one year (August 2020 to July 2021).

In addition to NGS and RT-qPCR variant concordance of the 78 samples, the results of this study reveal significantly reduced diversity of SARS-CoV-2 variants from July 2020 to August 2021. We detected 8 lineages among the 11 samples tested from July 2020 compared to a single Delta variant lineage with three sublineages (Delta) among 67 samples collected in August 2021 (Figure 2). These findings are important for understanding the evolution of SARS-CoV-2 variants in Texas (Figure 2) and support other studies showing the predominance and infectivity of the Delta variant [28].

## 4. Discussion

The emergence of new SARS-CoV-2 variants with higher infection rates and morbidity continues to cause the global scientific community concern. To manage further transmission and control of infection, genomic surveillance is important for the identification and tracking of novel variants. NGS is a very useful tool for identifying new strains of COVID-19 and other infectious pathogens. NGS can be used to detect novel pathogenic mutations and can also be used to determine the rate of pathogen evolution.

Although NGS is the most reliable method for detecting mutations in SARS-CoV-2, the methodology is not practically applicable for large-scale surveillance, particularly in resource-limited settings. Factors like continuous validation studies, logistic challenges, database validity, cost-benefit analysis, and high technical expertise make the implementation of NGS in routine clinical settings difficult. Comparatively, RT-qPCR—a gold standard for diagnosing SARS-CoV-2—is a method that can be extended for variant detection and monitoring in clinical settings. Although the cost of sequencing has plummeted in the last decade, and $1000 human genome is indeed a reality, the capital investment of the instrument (Illumina NovSeq) alone is ∼a million USD prior to any sequencing application. COVID-19 genome sequencing cost ranges from $100 to $400 (COGS [Cost of Goods] only). However, the laboratory must batch 1000s of samples to achieve the lowest cost. The fastest practically attainable turnaround time is ∼24 hrs for low throughput sequencing platforms which have the highest per sample cost. But RT-qPCR can be performed within a few hours and per sample costs (COGS: $5-$10) are a fraction of NGS. RT-qPCR also results in an easy-to-interpret numerical value (Ct value) compared to the complex NGS output (FASTQ files) requiring additional resources and time for analysis.

Accordingly, this study examined two commercially available RT-qPCR assays for the detection of SARS-CoV-2 mutagenic variant and mutation detection and compared the results with NGS. Both assays were able to detect L452R mutation with 100% (67/67; GT Molecular) and 94% (63/67; Thermo Fisher Scientific) accuracy when compared to NGS. While NGS is an essential tool for sequencing the entire genome and identification of new mutations, this study suggests that RT-qPCR can aptly serve as an easy-to-deploy, cost-effective, and time-sensitive solution for the detection of known mutations for mass surveillance. Likewise, this approach has been previously applied for surveillance of leprosy and identification of zoonotic transmission in the United States [29, 30]. The authors used NGS data to develop an algorithm for the classification of global variants and deployed RT-qPCR to understand the local transmission dynamics.

The results in this study are promising because the RT-qPCR lineage classification showed no mismatches when compared with the 21 sequenced samples that had raw data. Although these results are encouraging because of the low cost of scalability of SARS-CoV-2 mutation detection with RT-qPCR, other research studies using similar virus sequencing comparison methods have been less successful. Khan and Cheung [31] noted the presence of mismatches when comparing SARS-CoV-2 between RT-qPCR and sequencing data. Elaswad and Fawzy [32] also found this to be the case when comparing RT-qPCR assays with available SARS-CoV-2 genomes isolated from animals. Similarly, Hoang et al. [33] noted missed detection with RT-qPCR assays for influenza A (H1) when compared with sequencing. Although these studies add some concern, it does appear strategic deployment of both NGS and RT-qPCR technologies for the discovery and monitoring of emerging SARS-CoV-2 mutations is likely to advance better strategies for epidemiological characteristics.

Even though the unavailability of the raw data (FASTQ files) from the 67 samples remains the limitation of the study, phylogenetic analysis of the 21 samples tested at Advanta Genetics was clustered as expected; all the VOC and non-VOC samples were grouped appropriately. This suggests a good potential for the use of RT-qPCR approaches in the detection of preidentified mutations and possibly application in low-cost surveillance of known variants. Importantly, this study does not suggest the RT-qPCR as a replacement for NGS because RT-qPCR assays utilized in this study were designed to target only a few amino acid motifs compared to NGS, which covers a wider breadth of the virus genome.

## 5. Conclusion

There are two important takeaways from this study. First, the NGS data provided further evidence of the rapid evolution of SARS-CoV-2 lineages including the highly transmissible Delta variant in the East Texas region and suggests the continued threat of COVID-19. This finding is consistent with other research and further supports the need for rapid, cost-effective monitoring of variant mutations. Second, the current study endorses the potential of RT-qPCR assays as a solution for more accessible variant monitoring. The data showed concordance with RT-qPCR and NGS analysis for specific SARS-CoV-2 lineages and characteristic mutations. Thus, the deployment of RT-qPCR testing for the detection of known SARS-CoV-2 variants may be extremely beneficial.

The key difference between the NGS and RT-qPCR is discovery power, scalability, and throughput. Both technologies are reliable and highly sensitive. RT-qPCR can detect only known sequences with help of specific probes and primers. In contrast, NGS does not need prior information about the sequence, but NGS is less cost-effective for low target numbers and is a time-consuming method. NGS can detect thousands of targeted regions with single-base resolution. RT-qPCR is cost-effective, and its familiar workflow made the detection of a limited set of variants and low target numbers easy [34]. Accordingly, is it suggested that RT-qPCR is a quick and cost-effective alternative to sequencing for screening known mutations of SARS-CoV-2 for clinical and epidemiological interest, especially in developing countries where COVID-19 diagnostic centers are limited by regional sequencing laboratories for screening the mutations in the SARS-CoV-2 clinical samples. The findings in this study depict great potential for RT-qPCR to be an effective strategy offering several epidemiological advantages.

## Figures and Tables

**Table 1:** World Health Organization (WHO) designated variants of concern (VOC) [11].

WHO label	Alpha	Beta	Gamma	Delta	Omicron	
Pango lineage	B.1.1.7	B.1.351	P.1	B.1.617.2	B.1.1.529	
Classifying Mutation(s)	Δ69/70	K417N	K417N/T	T19R	A67V	E484A
	Δ144Y	E484K	E484K	(G142D^*∗*^)	Δ69-70	Q493K
	(E484K^*∗*^)	N501Y	N501Y	Δ156	T95I	G496S
	(S494P^*∗*^)	D614G	D614G	Δ157	G142D	Q498R
	N501Y			R158G	Δ143–145	N501Y
	A570D			L452R	Δ211	Y505H
	D614G			T478K	L212I	T547K
	P681H			D614G	ins214EPE	D614G
				P681R	G339D	H655Y
				D950N	S371L	N679K
					S373P	P681H
					S375F	N764K
					K417N	D796Y
					N440K	N856K
					G446S	Q954H
					S477N	N969K
					T478K	L981F

^*∗*^Detected in some sequences but not all.

**Table 2:** Data of representative samples (*n* = 21) sequenced and classified into Delta and non-VOC variants using NGS and RT-qPCR assays.

Sample	COVID-PCR (Ct values)		% of non-N bases (coverage≥10x)	Median coverage	COVID lineage by NGS		Variant-specific PCR (Ct values)				Lineage by RT-qPCR
							(GT molecular)		(Thermo fisher scientific)		
	N1	N2			Next clade	Pango	L452R	T478K	L452R	P681R	
NTC	Negative	Negative			N/A	N/A	Neg	Neg	Neg	Neg	N/A
665588	12.99	11.9	88.15	58	21A (elta)	AY.25	18.5	Neg	20.88	25.97	Delta
664789	28.72	27.78	99.40	221	21A (Delta)	AY.3	31.81	32.6	33.97	34.37	Delta
665660	16.71	15.64	98.45	116	21A (Delta)	AY.3	20.18	24.98	24.49	26.58	Delta
664822	26.3	25.24	93.87	103	21A (Delta)	AY.3	20.55	Neg	22.18	24.87	Delta
666013	14.5	13.98	99.36	217	21A (Delta)	B.1.617.2	22.72	33.27	27.8	28.78	Delta
665426	15.98	14.44	98.14	128	21A (Delta)	AY.3	18.69	25.39	23.92	25.52	Delta
21	21.41	21.71	100.0	1340	20C	B.1.243	Neg	Neg	Neg	Neg	Non-VOC
22	25.3	26.82	100.0	2380	19A	B.1.574	Neg	Neg	Neg	Neg	Non-VOC
23	25.35	25.04	100.0	1276	20C	B.1.574	Neg	Neg	Neg	Neg	Non-VOC
24	28.74	29.52	100.0	2618	20A	B.1.2	Neg	Neg	Neg	Neg	Non-VOC
25	19.86	20.43	100.0	229	20A	B.1	Neg	Neg	Neg	Neg	Non-VOC
26	17.33	17.64	100.0	1477	20C	B.1.234	Neg	Neg	Neg	Neg	Non-VOC
27	20.77	21.68	100.0	1092	20A	B.1.126	Neg	Neg	Neg	Neg	Non-VOC
28	19.98	20.58	100.0	1614	20C	B.1.602	Neg	Neg	Neg	Neg	Non-VOC
29	26.99	27.61	100.0	3734	20A	B.1	Neg	Neg	Neg	Neg	Non-VOC
210	33.9	32.64	100.0	1531	20C	B.1	Neg	Neg	Neg	Neg	Non-VOC
211	32.66	31.53	100.0	2485	20C	B.1.564	Neg	Neg	Neg	Neg	Non-VOC
212	33.19	35.37	100.0	2019	21A (Delta)	B.1.617.2	33.27	27.8	29.99	26.72	Delta
213	32.29	32.86	100.0	1888	21A (Delta)	AY.3	25.39	23.92	22.25	29.92	Delta
214	25.13	25.77	100.0	2618	21A (Delta)	B.1.617.2	32.32	33.12	24.93	31.56	Delta
Wuhan-hu1	25.26	25.89	100.00	3236	19B	A	Neg	Neg	Neg	Neg	Non-VOC

**Table 3:** Comparative detection of VOC classifying mutations with RT-qPCR and NGS-based approaches. ^*∗*^Variant determined by NGS (see Appendix for method).

Evaluation of Q-PCR-based SARS-CoV-2 variant detection solutions					VOC (*n* = 67)		Non-VOC (*n* = 11)	
					PCR	NGS	PCR	NGS
Manufacturer	Catalog #	Reaction	Targets	Variants (WHO label) harboring this mutation	0	0	0	0
GT molecular fort collins, CO, USA	100180	Reaction-1	N501Y	Alpha (B.1.1.7), beta (B.1.351), gamma (P.1)	0	0	0	0
			Del69-70	Alpha (UK, B.1.1.7)	0	0	0	0
		Reaction-2	N501Y	Alpha (B.1.1.7), Beta (B.1.351), gamma (P.1)	0	0	0	0
			E484K	Beta (B.1.351), gamma (P.1)	1	1	0	0
			K417N	Beta (B.1.351), Delta plus	0	0	0	0
			K417T	Gamma (P.1)	0	0	0	0
		Reaction - 3	L452R	Epsilon (B.1.427/B.1.429), Delta (B.1.617.2)	67	67	0	0
			T478K	Delta (B.1.617.2)	62	64	0	0
	100172	Reaction-1	L452R	Epsilon (B.1.427/B.1.429), Delta (B.1.617.2)	67	67	0	0
			T478K	Delta (B.1.617.2)	62	64	0	0

Thermo fisher scientific Inc., Waltham, MA, USA	A51819	Reaction-1	L452R	B.1.617, B.1.617.1, Delta (B.1.617.2), B.1.617.3, B.1.429	63	67	0	0
	A51822	Reaction-2	P681R	B.1.617.1, B.1.617.2, B.1.617.3	55	64	0	0

## Data Availability

The authors confirm that the data supporting the findings of this study are available within the article. Raw data (Genome Sequences) of this study are available on GISAID (EPI_ISL_8729878, EPI_ISL_8729889, EPI_ISL_8729877, EPI_ISL_8729888, EPI_ISL_8729879, EPI_ISL_8729874, EPI_ISL_8729885, EPI_ISL_8729884, EPI_ISL_8729876, EPI_ISL_8729887, EPI_ISL_8729875, EPI_ISL_8729886, EPI_ISL_8729881, EPI_ISL_8729892, EPI_ISL_8729880, EPI_ISL_8729891, EPI_ISL_8729883, EPI_ISL_8729882, EPI_ISL_8729890, EPI_ISL_8719355,EPI_ISL_8629671, EPI_ISL_8629670, EPI_ISL_8629673, EPI_ISL_8629672, EPI_ISL_8629675, EPI_ISL_8629674, EPI_ISL_8629666, EPI_ISL_8629676, EPI_ISL_8629668, EPI_ISL_8629667, EPI_ISL_8629669, EPI_ISL_8428058).

## Appendix

### A. RT-qPCR Primer and Probe Design

N1 Gene: 2019-nCoV_N1-Forward Primer—GACCCCAAA ATCAGCGAAAT; 2019-nCoV_N1-Reverse Primer-TCTGGTTACTGCCAGTTGAAT CTG; 2019-nCoV_N1- Probe- FAM-ACCCCGCATTACGTTTGGTGGACC-BHQ1. N2 Gene: 2019-nCoV_N2- Forward Primer- TTACAAACATTGGCCGCAAA; 2019-nCoV_N2- Reverse Primer—GCGCGACATTCCGAAGAA; 2019-nCoV_N2-Probe- FAM-ACAATTTGCCCCCAGCGCTTCAG-BHQ1.

## Supplementary Materials

Supplementary Materials Supplementary Table 1: detailed comparative analysis of existing SARS-CoV-2 variant detection using the Next-Generation Sequencing and qRT-PCR-based approaches.

## References

[1] El-Shehawi A. M., Alotaibi S. S., Elseehy M. M. (2020). Genomic study of COVID-19 corona virus excludes its origin from recombination or characterized biological sources and suggests a role for HERVS in its wide range symptoms. *Cytology and Genetics*.

[2] Morens D. M., Breman J. G., Calisher C. H. (2020). The origin of COVID-19 and why it matters. *The American Journal of Tropical Medicine and Hygiene*.

[3] CSSEGISandData (2021). COVID-19 data repository by the center for systems science and engineering (CSSE) at Johns Hopkins University. https://github.com/CSSEGISandData/COVID-19.

[4] Kevadiya B. D., Machhi J., Herskovitz J. (2021). Diagnostics for SARS-CoV-2 infections. *Nature Materials*.

[5] Ruch T. R., Machamer C. E. (2012). The coronavirus E protein: assembly and beyond. *Viruses*.

[6] Russo A., Minichini C., Starace M., Astorri R., Calo F., Coppola N. (2020). Current status of laboratory diagnosis for COVID-19: a narrative review. *Infection and Drug Resistance*.

[7] Cascella M., Rajnik M., Aleem A., Dulebohn S., Di Napoli R. (2021). Features, evaluation, and treatment of coronavirus (COVID-19). https://www.statpearls.com/ArticleLibrary/viewarticle/52171.

[8] Ibn-Mohammed T., Mustapha K. B., Godsell J. (2021). A critical analysis of the impacts of COVID-19 on the global economy and ecosystems and opportunities for circular economy strategies. *Resources, Conservation and Recycling*.

[9] Banoun H. (2021). Evolution of SARS-CoV-2: review of mutations, role of the host immune system. *Nephron*.

[10] (2021). Classification of omicron (B.1.1.529): SARS-CoV-2 variant of concern. https://www.who.int/news/item/26-11-2021-classification-of-omicron-(b.1.1.529)-sars-cov-2-variant-of-concern.

[11] (2022). SARS-CoV-2 variants of concern. UpToDate. https://www.uptodate.com/contents/image?imageKey=ID%2F131216.

[12] Sanger F., Nicklen S., Coulson A. R. (1977). DNA sequencing with chain-terminating inhibitors. *Proceedings of the National Academy of Sciences of the USA*.

[13] Sikkema-Raddatz B., Johansson L. F., De Boer E. N. (2013). Targeted next-generation sequencing can replace sanger sequencing in clinical diagnostics. *Human Mutation*.

[14] Motayo B. O., Oluwasemowo O. O., Olusola B. A. (2021). Evolution and genetic diversity of SARS-CoV-2 in Africa using whole genome sequences. *International Journal of Infectious Diseases*.

[15] Matthijs G., Souche E., Alders M. (2016). Guidelines for diagnostic next-generation sequencing. *European Journal of Human Genetics*.

[16] Lou E. G., Sapoval N., McCall C. (2022). Direct comparison of RT-ddPCR and targeted amplicon sequencing for SARS-CoV-2 mutation monitoring in wastewater. *Science of the Total Environment*.

[17] Wang H., Jean S., Eltringham R. (2021). Mutation-specific SARS-CoV-2 PCR screen: rapid and accurate detection of variants of concern and the identification of a newly emerging variant with spike L452R mutation. *Journal of Clinical Microbiology*.

[18] Bechtold P., Wagner P., Hosch S. (2021). Rapid identification of SARS-CoV-2 variants of concern using a portable peakPCR platform. *Analytical Chemistry*.

[19] https://www.ecdc.europa.eu/sites/default/files/documents/Methods-for-the-detection-and-identification-of-SARS-CoV-2-variants.pdf.

[20] Yaniv K., Ozer E., Shagan M. (2021). Direct RT-qPCR assay for SARS-CoV-2 variants of concern (Alpha, B.1.1.7 and Beta, B.1.351) detection and quantification in wastewater. *Environmental Research*.

[21] Smyth D. S., Trujillo M., Gregory D. A. (2022). Tracking cryptic SARS-CoV-2 lineages detected in NYC wastewater. *Nature Communications*.

[22] Hadfield J., Megill C., Bell S. M. (2018). Nextstrain: real-time tracking of pathogen evolution. *Bioinformatics*.

[23] Sievers F., Wilm A., Dineen D. (2011). Fast, scalable generation of high-quality protein multiple sequence alignments using clustal omega. *Molecular Systems Biology*.

[24] Kumar S., Stecher G., Tamura K. (2016). MEGA7: molecular evolutionary genetics analysis version 7.0 for bigger datasets. *Molecular Biology and Evolution*.

[25] Beck K. L., Seabolt E., Agarwal A. (2021). Semi-supervised pipeline for autonomous annotation of SARS-CoV-2 genomes. *Viruses*.

[26] Dereeper A., Guignon V., Blanc G. (2008). Phylogeny.fr: robust phylogenetic analysis for the non-specialist. *Nucleic Acids Research*.

[27] Bawono P., Heringa J., Russell D. J. (2014). PRALINE: a versatile multiple sequence alignment toolkit. *Multiple Sequence Alignment Methods*.

[28] Zhang M., Xiao J., Deng A. (2021). Transmission dynamics of an outbreak of the COVID-19 delta variant B.1.617.2—Guangdong province, China, may-june 2021. *China CDC Weekly*.

[29] Truman R. W., Singh P., Sharma R. (2011). Probable zoonotic leprosy in the southern United States. *New England Journal of Medicine*.

[30] Sharma R., Singh P., Loughry W. J. (2015). Zoonotic leprosy in the southeastern United States. *Emerging Infectious Diseases*.

[31] Khan K. A., Cheung P. (2020). Presence of mismatches between diagnostic PCR assays and coronavirus SARS-CoV-2 genome. *Royal Society Open Science*.

[32] Elaswad A., Fawzy M. (2021). Mutations in animal SARS-CoV-2 induce mismatches with the diagnostic PCR assays. *Pathogens*.

[33] Hoang P., Nguyen H., Tran H. (2019). Missed detections of influenza A (H1)pdm09 by real-time RT-PCR assay due to haemagglutinin sequence mutation, december 2017 to march 2018, northern Viet Nam. *Western Pacific Surveillance and Response Journal*.

[34] Ozsolak F., Milos P. M. (2011). RNA sequencing: advances, challenges and opportunities. *Nature Reviews Genetics*.

