# Supplementary material for: Confirming Multiplex RT-qPCR Use in COVID-19 with Next-Generation Sequencing: Strategies for Epidemiological Advantage
Source: Glob Health Epidemiol Genom. 2022 Jul 30;2022:2270965. doi: 10.1155/2022/2270965 (PMC9339135; doi:10.1155/2022/2270965)
Supplement: Supplementary Materials — Supplementary Table 1: detailed comparative analysis of existing SARS-CoV-2 variant detection using the Next-Generation Sequencing and qRT-PCR-based approaches. [file 2270965.f1.pdf]

Table-S1: Comparative analysis of existing SARS CoV-2 variant detection using the Next Generation Sequencing and qRT-PCR based approaches

Sr No	Lab ID					Evaluation of Q-PCR based SARS-CoV-2 variant detection solutions													
						VOC Varient PCR (GT Mol)						Delta Varient PCR (GT Mol)		Delta Varient PCR (Thermo)					
		Date of Collection	County	State	Postal	N501Y	Del69-70	N501Y	E484K	K417N	K417T	L452R	T478K	L452R	P681R	PANGO Lineage	WHO Name	Lineage Probability	Mean Coverage Depth
1	ab40213	2021-07-30	SMITH	TX	75709	Neg	Neg	Neg	Neg	Neg	Neg	22.72	33.27	27.8	28.78	B.1.617.2	Delta	100.0%	22127.2
2	665426	2021-07-29	HOOD	TX	76033	Neg	Neg	Neg	21.37	Neg	Neg	18.69	25.39	23.92	25.52	B.1.617.2	Delta	100.0%	37341.1
3	ab40238	2021-07-30	SMITH	TX	75762	Neg	Neg	Neg	Neg	Neg	Neg	27.27	32.32	33.12	**36.13**	B.1.617.2.3	Delta	100.0%	1809.3
4	665734	2021-07-29	BRAZOS	TX	77802	Neg	Neg	Neg	Neg	Neg	Neg	19.75	25.36	25.55	29.68	B.1.617.2	Delta	100.0%	35808.2
5	665448	2021-07-28	VAN ZANDT	TX	75147	Neg	Neg	Neg	Neg	Neg	Neg	16.73	21.04	21.93	26.9	B.1.617.2.3	Delta	100.0%	35788.0
6	666010	2021-07-30	JOHNSON	TX	76093	Neg	Neg	Neg	Neg	Neg	Neg	24.91	29.75	29.32	31.59	B.1.617.2	Delta	100.0%	47985
7	666044	2021-07-31	SMITH	TX	75702	Neg	Neg	Neg	Neg	Neg	Neg	19.87	29.59	25.88	29.93	B.1.617.2.3	Delta	100.0%	30345.7
8	664819	2021-07-28	SMITH	TX	75702	Neg	Neg	Neg	Neg	Neg	Neg	25.76	31.83	29.99	32.55	B.1.617.2.3	Delta	100.0%	15381.2
9	665664	2021-07-30	SMITH	TX	75702	Neg	Neg	Neg	Neg	Neg	Neg	16.88	25.45	22.25	27.06	B.1.617.2.3	Delta	100.0%	35543.9
10	665731	2021-07-29	WASHINGTON	TX	77833	Neg	Neg	Neg	Neg	Neg	Neg	19.62	25.41	24.93	27.53	B.1.617.2.3	Delta	100.0%	35032.8
11	665743	2021-07-30	BRAZOS	TX	77845	Neg	Neg	Neg	Neg	Neg	Neg	21.44	26.72	26.64	31.39	B.1.617.2	Delta	100.0%	41262.8
12	665337	2021-07-29	SMITH	TX	75702	Neg	Neg	Neg	Neg	Neg	Neg	24.84	29.92	25.8	30.2	B.1.617.2.3	Delta	100.0%	49623.0
13	665333	2021-07-29	SMITH	TX	75702	Neg	Neg	Neg	Neg	Neg	Neg	27.73	31.56	34.9	**37.08**	B.1.617.2.3	Delta	100.0%	2394.7
14	666007	2021-07-31	JOHNSON	TX	76058	Neg	Neg	Neg	Neg	Neg	Neg	19.2	24.15	23.8	26.32	B.1.617.2	Delta	100.0%	48201.1
15	666004	2021-07-31	HENDERSON	TX	75763	Neg	Neg	Neg	Neg	Neg	Neg	24.89	30.3	30.61	34.05	B.1.617.2	Delta	100.0%	6792.1
16	664792	2021-07-27	SMITH	TX	75702	Neg	Neg	Neg	Neg	Neg	Neg	23.66	29.47	27.99	30.58	B.1.617.2.3	Delta	100.0%	27506.0
17	665163	2021-07-28	TARRANT	TX	76117	Neg	Neg	Neg	Neg	Neg	Neg	18.99	25.9	22.73	25.21	B.1.617.2	Delta	100.0%	32888.9
18	ab40199	2021-07-30	SMITH	TX	75703	Neg	Neg	Neg	Neg	Neg	Neg	27.66	33	32	**36.83**	B.1.617.2	Delta	100.0%	6849.1
19	ab40215	2021-07-30	SMITH	TX	75707	Neg	Neg	Neg	Neg	Neg	Neg	18.9	23.48	24.47	29.01	B.1.617.2.3	Delta	100.0%	49129.7
20	665432	2021-07-29	JOHNSON	TX	76031	Neg	Neg	Neg	Neg	Neg	Neg	20.59	25.89	25.39	29.81	B.1.617.2	Delta	100.0%	67119.2
21	665415	2021-07-29	HOOD	TX	76049	Neg	Neg	Neg	Neg	Neg	Neg	22.45	28.26	27.36	31.27	B.1.617.2	Delta	100.0%	40157.8
22	666010	2021-07-30	JOHNSON	TX	76093	Neg	Neg	Neg	Neg	Neg	Neg	19.94	24.78	24.24	28.9	B.1.617.2	Delta	100.0%	47985.0
23	664829	2021-07-28	SMITH	TX	75702	Neg	Neg	Neg	Neg	Neg	Neg	24.99	28.93	31.93	34.41	B.1.617.2.3	Delta	100.0%	22329.0
24	664875	2021-07-27	BRAZOS	TX	77808	Neg	Neg	Neg	Neg	Neg	Neg	21	26.96	24.93	27.52	B.1.617.2	Delta	100.0%	47290.6
25	665961	2021-07-30	BRAZOS	TX	77845	Neg	Neg	Neg	Neg	Neg	Neg	19.24	27.88	23.01	25.28	B.1.617.2	Delta	100.0%	33387.6
26	ab40212	2021-07-30	SMITH	TX	75762	Neg	Neg	Neg	Neg	Neg	Neg	29.39	34.02	33.82	**38.46**	B.1.617.2	Delta	100.0%	1288.2
27	665674	2021-07-30	SMITH	TX	75702	Neg	Neg	Neg	Neg	Neg	Neg	23.03	27.33	27.99	32.12	B.1.617.2.3	Delta	100.0%	57325.7
28	665670	2021-07-30	SMITH	TX	75702	Neg	Neg	Neg	Neg	Neg	Neg	27.44	32.2	32.8	**35.94**	B.1.617.2.3	Delta	100.0%	10491.7
29	ab40159	2021-07-30	SMITH	TX	75703	Neg	Neg	Neg	Neg	Neg	Neg	24.2	30.53	28.7	31.04	B.1.617.2	Delta	100.0%	43664.1
30	666013	2021-07-31	JOHNSON	TX	76031	Neg	Neg	Neg	Neg	Neg	Neg	20.55	**Neg**	22.18	24.87	B.1.617.2	Delta	100.0%	69088.9
31	664812	2021-07-28	SMITH	TX	75702	Neg	Neg	Neg	Neg	Neg	Neg	22.66	28.21	26.75	31.78	B.1.617.2.3	Delta	100.0%	54609.4
32	664822	2021-07-28	SMITH	TX	75702	Neg	Neg	Neg	Neg	Neg	Neg	30.32	**37.99**	33.49	**35.72**	B.1.617.2.3	Delta	100.0%	2860.5
33	665205	2021-07-29	HIDALGO	TX	78574	Neg	Neg	Neg	Neg	Neg	Neg	17.73	21.9	20.34	23.49	B.1.617.2	Delta	100.0%	35647.4
34	665660	2021-07-30	SMITH	TX	75702	Neg	Neg	Neg	Neg	Neg	Neg	20.18	24.98	24.49	26.58	B.1.617.2.3	Delta	100.0%	54823.9
35	665341	2021-07-29	SMITH	TX	75702	Neg	Neg	Neg	Neg	Neg	Neg	29.88	**37**	33.68	**38.52**	B.1.617.2.3	Delta	100.0%	4566.0
36	665411	2021-07-28	HOOD	TX	76048	Neg	Neg	Neg	Neg	Neg	Neg	28.31	29.74	31.66	33.16	B.1.617.2	Delta	100.0%	61671.5
37	665880	2021-07-30	TARRANT	TX	76137	Neg	Neg	Neg	Neg	Neg	Neg	19.67	25.7	23.76	26.92	B.1.617.2	Delta	100.0%	71332.2
38	666039	2021-07-30	BRAZOS	TX	77845	Neg	Neg	Neg	Neg	Neg	Neg	19.79	25.97	24.64	26.98	B.1.617.2.3	Delta	100.0%	70771.4
39	664789	2021-07-27	SMITH	TX	75702	Neg	Neg	Neg	Neg	Neg	Neg	31.81	32.6	33.97	34.37	B.1.617.2.3	Delta	100.0%	341.6
40	665225	2021-07-28	SMITH	TX	75757	Neg	Neg	Neg	Neg	Neg	Neg	25.48	31.19	29.5	30.84	B.1.617.2	Delta	100.0%	57742.9
41	665678	2021-07-30	SMITH	TX	75702	Neg	Neg	Neg	Neg	Neg	Neg	28.16	35.22	33.16	34.78	B.1.617.2.3	Delta	100.0%	2096.2
42	665676	2021-07-30	SMITH	TX	75702	Neg	Neg	Neg	Neg	Neg	Neg	20.85	25.19	24.67	29.86	B.1.617.2.3	Delta	100.0%	33664.1
43	665588	2021-07-29	SMITH	TX	75771	Neg	Neg	Neg	Neg	Neg	Neg	18.5	**Neg**	20.88	25.97	B.1.617.2.25	Delta	100.0%	77387.8
44	665667	2021-07-30	SMITH	TX	75702	Neg	Neg	Neg	Neg	Neg	Neg	24.98	29.05	29.91	33.41	B.1.617.2.3	Delta	100.0%	40148.8
45	ab40145	2021-07-31	SMITH	TX	75706	Neg	Neg	Neg	Neg	Neg	Neg	22.79	26.7	26.67	30.51	B.1.617.2	Delta	100.0%	57736.7
46	666038	2021-07-31	SMITH	TX	75702	Neg	Neg	Neg	Neg	Neg	Neg	33.65	**Neg**	**38.28**	**39.97**	B.1.617.2.3	Delta	100.0%	3672.1
47	664813	2021-07-28	SMITH	TX	75702	Neg	Neg	Neg	Neg	Neg	Neg	21.99	27.45	26.14	28.88	B.1.617.2.3	Delta	100.0%	54978.4
48	665213	2021-07-29	TARRANT	TX	76020	Neg	Neg	Neg	Neg	Neg	Neg	23.21	28.36	27.35	28.35	B.1.617.2	Delta	100.0%	66473.2
49	ab43432	2021-07-30	HARRISON	TX	75670	Neg	Neg	Neg	Neg	Neg	Neg	25.17	31.64	29.92	32.32	B.1.617.2.3	Delta	100.0%	11141.2
50	665659	2021-07-30	SMITH	TX	75702	Neg	Neg	Neg	Neg	Neg	Neg	29.63	33.82	**35.52**	**38.89**	B.1.617.2.3	Delta	100.0%	1029.7
51	665476	2021-07-29	HARRIS	TX	77041	Neg	Neg	Neg	Neg	Neg	Neg	29.8	33.99	**35.84**	**36.09**	B.1.617.2	Delta	100.0%	2425.0
52	ab40209	2021-07-30	SMITH	TX	75762	Neg	Neg	Neg	Neg	Neg	Neg	26.36	31.82	31.14	32.68	B.1.617.2	Delta	100.0%	9087.8
53	ab40131	2021-07-31	SMITH	TX	75704	Neg	Neg	Neg	Neg	Neg	Neg	18.69	20.67	27.79	28.14	B.1.617.2	Delta	100.0%	32246.4
54	666045	2021-07-31	SMITH	TX	75702	Neg	Neg	Neg	Neg	Neg	Neg	18.33	23.89	22.34	24.53	B.1.617.2.3	Delta	100.0%	36172.8
55	664778	2021-07-27	SMITH	TX	75702	Neg	Neg	Neg	Neg	Neg	Neg	28.21	33.7	33.89	**35.81**	B.1.617.2.3	Delta	100.0%	2849.4
56	665032	2021-07-28	JOHNSON	TX	76044	Neg	Neg	Neg	Neg	Neg	Neg	23.89	31.91	25.66	27.8	B.1.617.2.3	Delta	100.0%	22923.6
57	665658	2021-07-30	SMITH	TX	75702	Neg	Neg	Neg	Neg	Neg	Neg	19.42	25.04	25.9	27.78	B.1.617.2.3	Delta	100.0%	29240.8
58	665340	2021-07-29	SMITH	TX	75702	Neg	Neg	Neg	Neg	Neg	Neg	26.71	31.81	30.25	32.2	B.1.617.2.3	Delta	100.0%	19843.7
59	665335	2021-07-29	SMITH	TX	75702	Neg	Neg	Neg	Neg	Neg	Neg	26.29	29.67	27.96	30.41	B.1.617.2.3	Delta	100.0%	36682.4
60	665602	2021-07-30	HENDERSON	TX	75763	Neg	Neg	Neg	Neg	Neg	Neg	26.07	31.54	32.17	32.65	B.1.617.2	Delta	100.0%	9208.1
61	665783	2021-07-30	SMITH	TX	75701	Neg	Neg	Neg	Neg	Neg	Neg	19.99	24.98	24.92	27.22	B.1.617.2	Delta	100.0%	29924.4
62	666043	2021-07-31	SMITH	TX	75702	Neg	Neg	Neg	Neg	Neg	Neg	21.97	26.5	27.87	29.29	B.1.617.2.3	Delta	100.0%	26266.6
63	664804	2021-07-28	SMITH	TX	75702	Neg	Neg	Neg	Neg	Neg	Neg	23.84	31.64	27.54	30.2	B.1.617.2.3	Delta	100.0%	24351.7
64	664807	2021-07-27	SMITH	TX	75702	Neg	Neg	Neg	Neg	Neg	Neg	33.15	**Neg**	**39.09**	**39.09**	B.1.617.2.3	Delta	100.0%	317.0
65	PreD 1	Aug-20	SMITH	TX	75703	Neg	Neg	Neg	Neg	Neg	Neg	Neg	Neg	Neg	Neg	B.1.243	Non-VOC	100.0%	1340
66	PreD 2	Aug-20	SMITH	TX	75703	Neg	Neg	Neg	Neg	Neg	Neg	Neg	Neg	Neg	Neg	B.1.574	Non-VOC	100.0%	2380
67	PreD 6	Aug-20	SMITH	TX	75703	Neg	Neg	Neg	Neg	Neg	Neg	Neg	Neg	Neg	Neg	B.1.574	Non-VOC	100.0%	1276
68	PreD 7	Aug-20	SMITH	TX	75703	Neg	Neg	Neg	Neg	Neg	Neg	Neg	Neg	Neg	Neg	B.1.2	Non-VOC	100.0%	2618
69	PreD 8	Aug-20	SMITH	TX	75703	Neg	Neg	Neg	Neg	Neg	Neg	Neg	Neg	Neg	Neg	B.1	Non-VOC	100.0%	229
70	PreD 10	Aug-20	SMITH	TX	75703	Neg	Neg	Neg	Neg	Neg	Neg	Neg	Neg	Neg	Neg	B.1.234	Non-VOC	100.0%	1477
71	PreD 12	Aug-20	SMITH	TX	75703	Neg	Neg	Neg	Neg	Neg	Neg	Neg	Neg	Neg	Neg	B.1.126	Non-VOC	100.0%	1092
72	PreD 13	Aug-20	SMITH	TX	75703	Neg	Neg	Neg	Neg	Neg	Neg	Neg	Neg	Neg	Neg	B.1.602	Non-VOC	100.0%	1614
73	PreD 14	Aug-20	SMITH	TX	75703	Neg	Neg	Neg	Neg	Neg	Neg	Neg	Neg	Neg	Neg	B.1	Non-VOC	100.0%	3734
74	PreD 15	Aug-20	SMITH	TX	75703	Neg	Neg	Neg	Neg	Neg	Neg	Neg	Neg	Neg	Neg	B.1	Non-VOC	100.0%	1531
75	PreD 16	Aug-20	SMITH	TX	75703	Neg	Neg	Neg	Neg	Neg	Neg	Neg	Neg	Neg	Neg	B.1.564	Non-VOC	100.0%	2485
76	PreD 18	Aug-20	SMITH	TX	75703	Neg	Neg	Neg	Neg	Neg	Neg	29.99	27.8	33.27	26.72	B.1.617.2.25	Delta	100.0%	2019
77	PreD 19	Aug-20	SMITH	TX	75703	Neg	Neg	Neg	Neg	Neg	Neg	22.25	23.92	25.39	29.92	B.1.617.2.3	Delta	100.0%	1888
78	PreD 20	Aug-20	SMITH	TX	75703	Neg	Neg	Neg	Neg	Neg	Neg	24.93	33.12	32.32	31.56	B.1.617.2	Delta	100.0%	2618
	Alpha -Ctrl					24.83	24.52	Neg	Neg	Neg	Neg	Neg	Neg	Neg	Neg	**B.1.1.7**	Alpha	NA	NA
	Beta -Ctrl					Pos	Neg	Neg	27.78	23.2	Neg	Neg	Neg	Neg	Neg	**B.1.351**	Beta	NA	NA
	Delta -Ctrl					Neg	Neg	Neg	37.73	Neg	Neg	Pos	Pos	Neg	Neg	**B.1.617.2**	Delta	NA	NA
	Gamma -Ctrl					Neg	Neg	24.84	Neg	23.97	Neg	Neg	Neg	Neg	Neg	**P.1**	Gamma	NA	NA
	Wuhan -Ctrl					Neg	Neg	Neg	Neg	Neg	Neg	Neg	Neg	Neg	Neg	A	Non-VOC	NA	NA
